# Update on Management of Non-proliferative Diabetic Retinopathy without Diabetic Macular Edema; Is There a Paradigm Shift?

**DOI:** 10.18502/jovr.v17i1.10175

**Published:** 2022-01-21

**Authors:** Amir Arabi, Ramin Tadayoni, Hamid Ahmadieh, Toktam Shahraki, Homayoun Nikkhah

**Affiliations:** ^1^Ophthalmic Research Center, Research Institute for Ophthalmology and Vision Science, Shahid Beheshti University of Medical Sciences, Tehran, Iran; ^2^Department of Ophthalmology, Torfeh Medical Center, Shahid Beheshti University of Medical Sciences, Tehran, Iran; ^3^Université de Paris, Ophthalmology Department, AP-HP, Lariboisière, Saint Louis and Fondation Adolphe de Rothschild Hospitals, Paris, France; ^4^Department of Ophthalmology, Labbafinejad Medical Center, Shahid Beheshti University of Medical Sciences, Tehran, Iran; ^5^Department of Ophthalmology, Imam Hossein Medical Center, Shahid Beheshti University of Medical Sciences, Tehran, Iran

**Keywords:** Diabetic Macular Edema, Management, Nonproliferative Diabetic Retinopathy, Paradigm Shift

## Abstract

Diabetic retinopathy (DR) is the major cause of visual impairment and blindness in the working-age population. Conventional management for nonproliferative diabetic retinopathy (NPDR) without diabetic macular edema (DME) is derived from the findings of the Early Treatment Diabetic Retinopathy Study (ETDRS). Although the ETDRS protocol basically includes observation, selected cases of severe NPDR may undergo scatter laser photocoagulation. Post-hoc analysis of recent trials has shown that patients with NPDR receiving intravitreal anti-vascular endothelial growth factor (anti-VEGF) for DME would experience improvement in the DR severity scale (DRSS). In addition, recent randomized trials (PANORAMA and Protocol W) have revealed that early intervention with intravitreal aflibercept in eyes with moderately severe to severe NPDR is associated with significant improvement in DRSS and reduced vision-threatening complications of DR. Based on recent studies, it seems that the therapeutic approach to NPDR may undergo a substantial change and a paradigm shift toward considering early intervention with the administration of intravitreal anti-VEGF injections. However, the long-term results and the duration of adherence to anti-VEGF therapy for eyes with NPDR are not yet defined. It is also not apparent whether improvement in DRSS is a true disease modification. Studies showed that DRSS improvement is not associated with retinal reperfusion. In addition, DRCR.net Protocol W showed no visual acuity benefit with the early intravitreal aflibercept injection in moderate to severe NPDR as compared with performing observation plus intravitreal aflibercept applied only after progression to proliferative DR or vision-impairing DME. The cost–benefit ratio is also a challenge. Herein, we look at different aspects of early anti-VEGF application and discuss its pros and cons in the process of treating NPDR.

##  INTRODUCTION

According to the recent report of the International Diabetes Federation (IDF), about 400 million people live with diabetes mellitus (DM) worldwide; this prevalence is estimated to approach 600 million individuals by 2035.^[1]^ One of the most common microvascular complications of DM is diabetic retinopathy (DR), which is reported to be the leading cause of visual impairment in the working-age population.^[2,3]^ Diabetic retinopathy is classically categorized into two types: nonproliferative diabetic retinopathy (NPDR) and proliferative diabetic retinopathy (PDR). Diabetic macular edema (DME) is another important manifestation of DR, which may be experienced across all DR severity stages. While approaches to the patients with PDR or DME is straightforward, the therapeutic approach to NPDR patients with no DME has not been well established.

Conventional management for NPDR without DME, which is derived from the findings of the Early Treatment Diabetic Retinopathy Study (ETDRS)^[4]^ includes observation for mild and moderate NPDR. Most cases of severe NPDR are also followed closely; however, selected cases may undergo scatter laser photocoagulation. Post-hoc analysis of recent trials has shown that patients with NPDR receiving intravitreal anti-vascular endothelial growth factor (anti-VEGF) drugs for DME would experience amelioration in the DR severity scale (DRSS).^[5,6]^ In addition, more recent randomized controlled trials (PANORAMA study and DRCR.net Protocol W) have revealed that early intervention with intravitreal injection of aflibercept in eyes with moderately severe to severe NPDR may be associated with significant improvement in DRSS and reduced vision-threatening complications although the effect on visual acuity has not been significantly different compared to proper follow-up with timely treatment of complications.^[7,8]^ Based on recent studies, the therapeutic approach toward treating NPDR may undergo a significant change and paradigm shift to early intervention with intravitreal anti-VEGF injections that may substitute the conventional approach. However, the long-term results of anti-VEGF therapy for eyes with NPDR are not yet determined, and it is not clear how durable this approach will be and whether it is connected with enhanced visual functions and improved quality of life (QoL). The cost–benefit ratio is also a challenge that needs to be addressed. Herein, we review the different aspects of NPDR management and the early application of anti-VEGF therapy.

### Management of NPDR Without DME

Management of NPDR patients without DME involves all interventions that prevent occurrence of vision-threatening complications including PDR and DME. This goal can be achieved by both systemic and ocular interventions.

### Systemic management

#### Glycemic control

Control of hyperglycemia remains the basis of care in diabetic patients. Intensive glycemic control evaluated in two landmark trials, the Diabetes Control and Complications Trial (DCCT) and the UK Prospective Diabetes Study (UKPDS), assisted in reducing the risk of developing retinopathy and slowing the DR progression in both type 1 and type 2 DM^[9,10]^ These results have been supported in other studies.^[11,13]^ As an observation in the DCCT and UKPDS, the people in the early intensive glycemic control group had a significantly lower risk for long-term retinopathy progression and microvascular disorders regardless of the glycemic condition in the later course of the diabetes.^[14,15]^ The American Diabetes Association (ADA) recommends a hemoglobin A1c (HbA1c) of < 7%, with the recommendation of adjustment on an individual basis to avoid probable complications, such as hypoglycemia.^[16]^

#### Blood pressure control

Several studies have investigated the role of blood pressure regulation in the incidence and progression of DR. Some of these studies have demonstrated a positive effect from the intensive control of blood pressure, whereas no beneficial effect on the incidence and progression of DR has been observed in others.^[17,18,19,20]^ Generally, blood pressure control has been recommended as a principal part of the standard care in diabetic patients, primarily because of its known beneficial effect on macro-vascular complications of DM rather than for its effect on DR.^[21]^ However, blood pressure control may also reduce the damage to endothelial cells, through which slowing of DR progression may be achieved.^[22]^ The available evidence does not support the idea that blood pressure control alone can inhibit or slow the progression of DR.^[23]^

#### Control of hyperlipidemia

The effects of dyslipidemia on DR incidence and progression have been controversial. In a new meta-analysis, no significant difference in lipid profile was observed between patients with and without DR.^[24]^ However, Sankara Nethralaya Diabetic Retinopathy Epidemiology and Molecular Genetic Study (SN-DREAMS II) reported a threefold increase in the risk of DR progression to PDR stage in patients with higher triglycerides levels.^[25]^ The ACCORD Eye Study has confirmed that fenofibrate benefits patients with DR.^[26]^ Furthermore, the FIELD study confirmed that fenofibrate could prevent the progression of DR independent of serum lipids' levels.^[27]^ It is postulated that the role of fenofibrate is more effective via iron chelation rather than hyperlipidemia treatment. Given the role of iron in retinal damages via oxidative pathways, iron chelation with fenofibrate may play a protective role in reducing retinal damage in DR.^[28]^ In the Collaborative Atorvastatin Diabetes Study and the Heart Protection Study, the progression of DR did not differ between patients treated with statins and those who received placebo.^[29,30]^

#### Miscellaneous systemic risk factors

Anemia is considered a risk factor of the microvascular complications of diabetic patients.^[31,32]^ Some studies have suggested that lower hemoglobin levels may be linked to progression of DR.^[33,34]^ Dietary modification, regular monitoring of anemia, and treatment with supplements may stop the progression of DR.^[35]^

A meta-analysis has revealed an association between vitamin D deficiency and increased risk of DR in type 2 DM.^[36]^ Recently, it has been postulated that vitamin D3 exerts protective effects against retinal cell apoptosis and vascular damage in DR patients via an anti-inflammatory mechanism.^[37]^

A new meta-analysis discovered that the risk of DR was greater in smokers with type 1 diabetes; while in those patients who suffered with type 2 diabetes, the risk of PDR significantly decreased in smokers in comparison with nonsmokers.^[38]^ In the Wisconsin Epidemiologic Study of Diabetic Retinopathy (WESDR), smoking was not significantly associated with the progression of DR in a 4-year and 10-year follow-up period.^[39]^ Although the relationship between smoking and progression of DR remains inconclusive, there is evidence that suggests that smoking encourages macro-vascular complications associated with DM. As a result, it is recommended that patients who suffer with DM be strongly advised to cease smoking.

### Ophthalmic management

#### Laser photocoagulation for NPDR 

As DR reaches proliferative stage, retinal photocoagulation is applied to preserve the vision. This indication was derived from the presentation of two landmark studies, Diabetic Retinopathy Study (DRS) and ETDRS, where the findings convinced ophthalmologists to reach consensus on laser photocoagulation as a gold standard procedure for high-risk PDR (HRPDR).^[40]^ Nevertheless, the question remains: Can retinal photocoagulation for patients with nonproliferative stages of DR decrease the risk of visual impairment by preventing the progression to PDR stage?

Patients with either PDR in at least one eye or severe NPDR in both eyes were included in the DRS.^[41]^ One eye of each patient was randomly assigned to laser photocoagulation and the second eye was considered as the control group. The two-year risk of severe visual loss in eyes with severe NPDR was 3.2% and 2.8% in the control and laser photocoagulation groups, respectively. The four-year rates were 12.8% and 4.3%, respectively. Although the researchers found that 50% of the eyes with severe NPDR who were in the control group developed new vessels in one year, after considering the small risk of severe visual loss in the control group and the possible side effects of laser photocoagulation they did not recommend laser photocoagulation for all NPDR patients. Nevertheless, the DRS offered laser photocoagulation in NPDR eyes in some instances: one eye of a patient with severe NPDR in both eyes, presence of severe retinal ischemia, when the patient was pregnant or there was coexisting disorders such as renal failure that might accelerate the course of DR.^[40]^

The ETDRS remains the only study addressing the question of the suitable time for starting laser photocoagulation.^[4]^ Patients with moderate to severe NPDR or early PDR were included. Early photocoagulation was randomly performed in one eye of each patient and the other eye was assigned to deferred photocoagulation. In the latter, patients underwent laser therapy when high-risk PDR was detected. In the deferred photocoagulation group who had severe NPDR, the rate of PDR development was 51%, 71%, and 79% in the first-, third-, and fifth-year visits, respectively.^[4]^ Compared to deferred photocoagulation, early photocoagulation decreased progression to high-risk PDR by 25% and 50% with full scatter and mild scatter photocoagulation, respectively.^[4]^ However, the rate at the five-year visit determined that severe visual loss was low and comparable among the study groups (2.6% and 3.7% in early and deferral photocoagulation groups, respectively).^[4]^

A recent survey built a Markov model to explore whether it would be cost-effective either to apply panretinal photocoagulation (PRP) at the NPDR stage or to wait until HRPDR developed.^[43]^ They found that earlier PRP at the severe NPDR stage was less costly and more effective than administering PRP to patients with high-risk PDR. It meant that fewer patients in the earlier PRP group progressed to more advanced stages of DR.

The most common complications of PRP are decreased visual field and exacerbation of macular edema.^[44,45]^ Fong et al have reported that visual field defects may occur in almost half of the treated patients, and the incidence is correlated with the intensity of the laser therapy.^[46]^ A recent study based on optical coherence tomography angiography (OCT-A) has reported that in laser-treated severe NPDR eyes, ocular blood flow is significantly reduced, which may be associated with decreased visual acuity in these patients.^[47]^

#### Anti-VEGF for NPDR

Intravitreal anti-VEGF therapy for NPDR is an evolving concept. Clinical data show that VEGF contributes to the pathogenesis of both NPDR and PDR.^[48,49]^ According to the results of retrospective studies, anti-VEGF treatment can improve the DRSS and reduce the rate of PDR development.^[50]^

####  Utilization of anti-VEGF in NPDR with DME

RISE and RIDE were two phase-III, double-blind randomized clinical trials of intravitreal ranibizumab versus sham in patients with DME. In an exploratory analysis of RISE and RIDE trials, among the eyes with baseline ETDRS severity level of 53 (severe NPDR) or less, monthly intravitreal ranibizumab injection administered for 24 months was associated with a ≥ 2-step improvement in DRSS in 47% of eyes, as compared to 6.8% in the sham group (*P* < 0.001).^[5]^ Furthermore, it has been noted that the cumulative probability of DR progression was 34% in the sham-treated patients and 11.2–11.5% in the ranibizumab-treated patients by month 24.^[5]^ In addition, it has been reported that intravitreal ranibizumab in patients of RISE and RIDE trials slowed the progression of retinal non-perfused areas.^[51]^

Similarly, in the VIVID-DME and VISTA-DME trials, a significantly greater proportion of patients treated with aflibercept (week 100: 34.9%) compared with those treated with laser (13%) achieved a ≥ 2 step DRSS improvement (*P* < 0.0001).^[52]^ In addition, the proportion of patients who developed PDR was significantly less in those who received intravitreal aflibercept as compared with the sham-treated group (week 100: 2.2% vs 9.1%, *P* < 0.0001).^[52]^

DRCR.net Protocol T compared the efficacy of intravitreal aflibercept, ranibizumab, and bevacizumab in the treatment of DME. Based on a post hoc analysis of the Protocol T, 25%, 22% and 31% of NPDR eyes receiving aflibercept, bevacizumab, and ranibizumab for DME demonstrated improvement in DRSS at two-year follow-up, respectively.^[53]^ There were no statistically significant differences among the three commercially available anti-VEGFs in terms of inducing the regression of DR.

#### Utilization of anti-VEGF in NPDR without DME

The PANORAMA study was the first randomized clinical trial evaluating the role of intravitreal aflibercept on DRSS and incidence of vision-threatening complications (PDR and/or anterior segment neovascularization) and center-involved (CI) DME in patients with moderately severe to severe NPDR without DME. In this phase-3 clinical trial, 402 patients were randomly assigned to sham versus aflibercept administered every 8 weeks after 5 monthly loading doses versus aflibercept administered every 16 weeks after 3 monthly loading doses. At two-year follow-up, the proportion of eyes with 2-step or more improvement in DRSS was 12.8%, 62.2%, and 50% in the sham group, every 16 weeks aflibercept group and every 8 weeks (converted to pro re nata [PRN] in the second year) aflibercept group, respectively (*P* < 0.001 for both).^[7]^ Furthermore, the proportion of eyes that developed vision-threatening complications and/or CI-DME were 50.4%, 16.3%, and 18.7% in the sham group, every 16 weeks and every 8 weeks aflibercept groups, respectively (*P* < 0.001 for both comparisons).

DRCR.net Protocol W is a phase-3 randomized clinical trial evaluating the role of intravitreal aflibercept (injected at baseline, months 1, 2, and 4; and after that every four months through to two years) versus sham in reducing vision-threatening complications in eyes with moderate to severe NPDR.^[8]^ While the primary endpoint was recently reported at two years, the patients will be followed-up to four years. The two-year risk of developing CI-DME with decreased visual acuity or PDR was 16.3% and 43.5% in the aflibercept and sham groups, respectively (adjusted hazard ratio, 0.32 [97.5% confidence interval { CI } , 0.21–0.5; *P* < 0.001]). DRSS improved ≥ 2-steps from baseline to year 2 in 44.8% and 13.7% of eyes receiving aflibercept and sham, respectively (adjusted odds ratio, 5.91 [97.5% CI, 3.19–10.95; *P* < 0.001]).

Monte Carlo simulation of a real-world cohort of treatment-naive patients with NPDR from the IBMⓇ ExplorysⓇ database suggested that severe NPDR treatment with anti-VEGF would significantly decrease the probability of progression to PDR by 51.7% at the five-year follow-up period. Furthermore, the incidences of sustained blindness in severe NPDR patients were reduced with anti-VEGF therapy by 57.7% over a 10-year follow-up period.^[54]^

The phase-2 BOULEVARD trial compared the efficacy of Faricimab, a bispecific antibody, inhibiting both VEGF-A and angiopoietin-2, with ranibizumab in the treatment of patients with DME.^[55]^ At the six-month follow-up period, among the patients who were treatment-naïve, 2-steps or greater improvement in the DRSS was achieved in 12.2%, 27.7%, and 38.6% of eyes in the 0.3 mg ranibizumab, 1.5 mg Faricimab, and 6 mg Faricimab groups, respectively.^[55]^

##  DISCUSSION

Conventional management of NPDR without DME included observation along with controlling the systemic condition. The ETDRS showed that in one year, 26% of eyes with moderately severe NPDR and 52% of eyes with severe NPDR in the deferred photocoagulation group would progress to PDR, a vision-threatening complication of DR. The rate of progression to PDR reached 66% and 75–81% at the five-year follow-up period.^[42]^ Recently, there has been increasing evidence that anti-VEGF treatments would improve DRSS and decrease the risk of vision-threatening complications such as PDR and DME,^[7,8]^ raising an important question: Is it recommended to target the DR at the nonproliferative stage by administering intravitreal anti-VEGF injections to prevent the disease progression and reduce the risk of vision threatening complications? There are pros and cons for this evolving approach.

### Pros of Using Anti-VEGF in NPDR Without DME

There is increasing evidence in favor of administering intravitreal anti-VEGF injection in nonproliferative stages of DR without DME. The PANORAMA study showed that intravitreal aflibercept reduces the risk of vision-threatening complications by 77% and 83% in every 16 weeks and every 8 weeks (PRN in the second year) groups, respectively, as compared with the sham group at 100 weeks.^[7]^ At the end of the second year, data were also emphasized in the DRCR.net W protocol, where the risk of vision-threatening complications was 16.3% and 43.5% for the aflibercept and the sham groups, respectively.^[8]^

Extension of non-perfusion areas is the major pathology in DR. Diabetic retinopathy leads to upregulation of VEGF and contributes to further progression of non-perfusion areas as a vicious cycle. It was demonstrated that anti-VEGF therapy slows the development and progression of retinal non-perfusion in patients with DME.^[51]^ However, a small interventional cohort with short term follow-up showed that anti-VEGF induced improvement of DRSS could occur without any retinal reperfusion.^[56]^

Epidemiological studies have shown that DR has an adverse effect on the quality of life (QoL). A recent longitudinal and observational study showed that QoL significantly decreases with aggravation of DR severity from mild NPDR to PDR.^[57]^ Furthermore, a cross-sectional study showed that vision-related functional burden is significantly greater in patients with severe NPDR or PDR versus those with no retinopathy.^[58]^

Longitudinal population-based studies have shown that more advanced DR at diagnosis is associated with higher risk of developing sustained blindness.^[59]^ Kaplan-Meier's analysis of a recent epidemiological study has shown that eyes with moderate NPDR, severe NPDR, and PDR were 2.6, 3.6, and 4 times, respectively, more likely to develop sustained blindness, as compared to eyes with mild NPDR, after two years of follow-up.^[59]^

### Cons of Using Anti-VEGF in NPDR Without DME

At the end of two-year follow-up in the PANORAMA study, 49.6% of eyes in the sham group did not develop vision-threatening complications and/or CI-DME.^[7]^ This shows that nearly half of the patients who have NPDR will not progress to PDR or develop DME despite not receiving any intraocular injection.

Some complications have been reported regarding the use of intravitreal anti-VEGF injections. Common complications are the incidence of floaters and the rise of IOP.^[60,61]^ The most devastating complication is infectious endophthalmitis. The prevalence of endophthalmitis following intravitreal injections is estimated to be 0.01–0.26%.^[62]^

It is not clear whether intravitreal anti-VEGF for severe NPDR can be associated with enhanced visual function and improved QoL. DRCR.net Protocol W, during a two-year period, showed no visual benefit of the preventive intravitreal aflibercept treatment in eyes with moderate to severe NPDR as compared with those eyes that underwent observation plus aflibercept which was administered only after progression to PDR or vision-impairing CI-DME. The mean change of visual acuity was –0.9 and –2.0 ETDRS letters in aflibercept and sham groups, respectively (*P* = 0.47).^[8]^

The long-term real-world benefits of anti-VEGF therapy for eyes with NPDR are not yet determined. It is not clear how durable the treatment is and how long the patients should receive anti-VEGF treatment. In the second year of the PANORAMA study, those patients who initially received aflibercept every eight weeks transitioned to PRN. Concomitantly the rate of 2-steps or more improvement in DRSS reduced from 79.9% to 50%.^[7]^ Furthermore, in the RISE/RIDE open label extension (OLE) study, nearly 40% of eyes that did not receive any more ranibizumab injections during the OLE experienced 2-steps or more worsening in the DRSS.^[63]^

It is not apparent whether improvement in the DRSS following intravitreal injections of anti-VEGFs is a true disease modification. In a case series by Couturier et al, no reflow of vessels or reperfusion of capillary bed was found in non-perfusion areas using ultra-widefield (UWF) fluorescein angiography (FA) and swept-source widefield (SS-WF) OCT-A in eyes with DR after 3 anti-VEGF injections.^[64]^ In addition, Bonnin et al showed that after administering anti-VEGF injections in DR eyes, the improvement in the DRSS score based on color fundus photograph could occur without retinal reperfusion on UWF FA.^[65]^ In an OLE of RISE/RIDE study, it was shown that patients with anti-VEGF injection induced moderate NPDR were more prone to DR progression compared to patients with moderate NPDR at enrollment who were randomized to the sham group.^[63]^

The cost–benefit ratio is also a challenge that needs to be addressed. Answering this question requires more time and further studies.

### Possible Effects of VEGF-independent Drugs on NPDR Course

Inhibition of the VEGF independent pathways may also affect the course of DR. There is some evidence that angiopoietin/Tie2 and Rho-associated kinase (ROCK) play an influential role in retinal perfusion. Inhibition of angiopoietin 2 may enhance the effects of VEGF inhibition in improving the DRSS.^[55]^

Expression of Rho-associated kinase (ROCK) is increased in diabetic eyes and activation of ROCK-1 induces focal retinal vasoconstriction and subsequent retinal ischemia.^[66,67]^ An in vivo study showed that fasudil (a specific ROCK inhibitor) decreased vasoconstriction, improved retinal flow, and could potentially reduce the retinal ischemia.^[67]^ Intravitreal ripasudil also decreased the retinal non-perfusion areas and improved retinal blood flow in a murine model of retinal vein occlusion.^[68]^ Ahmadieh et al reported that a combination of intravitreal bevacizumab and fasudil in eyes with persistent DME and macular ischemia was associated with significantly more visual improvement as compared with solely administering intravitreal bevacizumab. This significant visual improvement could be due to improved perfusion induced by the ROCK inhibitor.^[69]^ Further research is needed to determine the role of ROCK inhibitors in ameliorating diabetes-induced retinal microvascular damage and improving DRSS.

##  SUMMARY

The concept of slowing the progressive course of DR and preventing the vision-threatening complications of this potentially blinding disease may represent the initial sign of a paradigm shift from the observation, which has been the standard care for patients with NPDR to a new strategy comprising intravitreal anti-VEGF injections. However, there is not enough evidence supporting this paradigm shift at present. A new classification may help improving the management of NPDR based on recent progress in understanding of the pathophysiology and advances in treatment of DR and addresses the need to possible paradigm shift in the future.

##  Financial Support and Sponsorship

The authors declare that they did not receive any fund for the current manuscript or any research relevant to the present study.

##  Conflicts of Interest

The authors have no conflicts of interest to declare.
